# Physeal-Sparing Anterior Cruciate Ligament Reconstruction for Skeletally Immature Patients: All-Epiphyseal Technique Using Quadricep Tendon Autograft

**DOI:** 10.1155/2021/5519822

**Published:** 2021-04-13

**Authors:** Hatem B. Afana, Thomas Nau

**Affiliations:** ^1^Orthopaedic Department, King's College Hospital London, Dubai, UAE; ^2^MBRU-Mohamed Bin Rashid University, School of Medicine, Dubai, UAE

## Abstract

The anterior cruciate ligament (ACL) is a major stabilizing structure of the knee and one of the most common injured structures. The true incidence of ACL injury in children and adolescents is unknown, but recent studies suggest increased ACL injury rates, especially in the sports-participating population. The mechanism of injury, clinical examination, and diagnosis of ACL injury in children is the same as in adults. The main concerns in the management of pediatric ACL injuries are the open physes and the eventual long-term consequences of the ACL deficient knee. The ideal treatment strategy of pediatric ACL injuries is still controversial, because there is still no universal consensus for techniques, graft choices, and postoperative rehabilitation. We present a case of a 12-year-old male patient who underwent ACL reconstruction using an all-inside, physeal-sparing technique with a quadriceps tendon autograft and discuss the current treatment strategies.

## 1. Introduction

Anterior cruciate ligament (ACL) injury is the most common ligament injury in the knee; [1]. Most ACL injuries are noncontact sports-related [2], and it happens when the body is decelerating or during “dynamic knee valgus” movements. With an increasing number of children playing sports and improving the modalities to diagnose, the number of pediatrics cases diagnosed with ACL injury is becoming higher. Skiing, soccer, basketball, and football are the highest risk sports associated with ACL injury [3]. Young female athletes are more susceptible to ACL injury as they have a smaller intercondylar notch, smaller and more lax ligaments, and greater total valgus angle of the knee [4] (the female ACL: why is it more prone to injury?, [5]).

With no well-designed studies about the frequency of ACL injury in children and adolescents, the true incidence is usually underestimated, as most of the studies focus only on patients who undergo surgery and overlook the patients who did not [6]. A more recent study by Beck et al. shows an increase in incidences of ACL tears in pediatric patients over the last 20 years [7] [8].

Management of ACL tears in pediatrics remains controversial; advocates of operative management say there is an increased risk of meniscal damage, cartilage damage, and those children with nonoperative management will have longer sick leave from school. However, others prefer a nonoperative approach as there is a risk of growth disturbances and angular deformities after ACL reconstructive surgery. Also, there is no high-level evidence to suggest that ACL reconstruction prevents the risk of developing arthritis; although, studies have suggested that the risk of leg length difference or angular leg deviations is low after ACL reconstruction in children and adolescents [9], and cartilage and meniscal injuries that occur at the time of initial ACL rupture have been demonstrated to be the main predictors of arthritic changes [10].

## 2. Case Presentation

We present a surgical technique for an all-inside, all-epiphyseal ACL reconstruction using quadricep tendon autograft in skeletally immature patients and our postoperative rehabilitation protocol. This is an all-inside technique with the femoral tunnel drilled retrograde and the tibial tunnel drilled retrograde; both tunnels are entirely within the epiphyses. Fixation of the quadricep autograft is achieved with soft-tissue buttons on both the femur and tibia.

**Figure 1:**
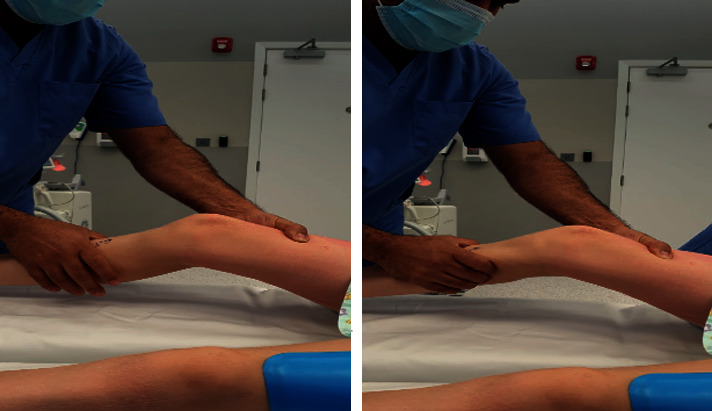
Lachman test.

**Figure 2:**
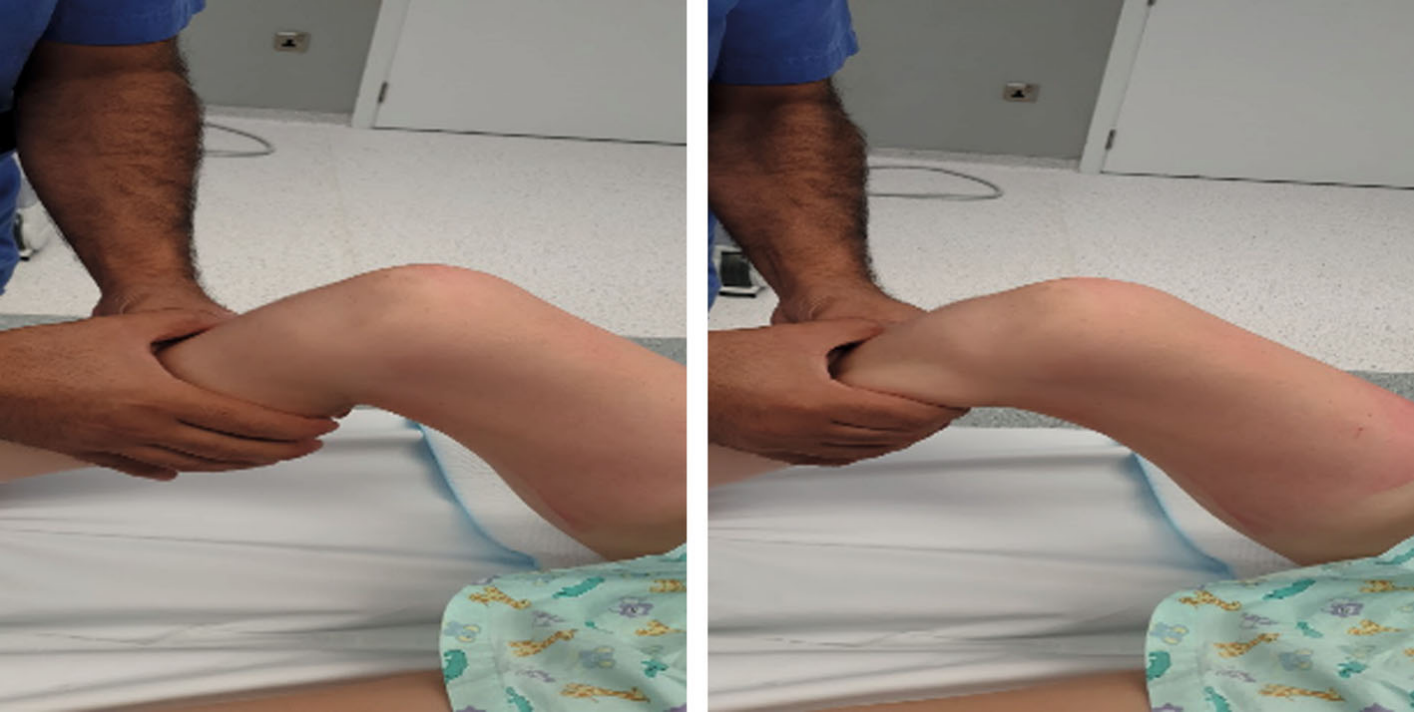
Anterior drawer test.

A previously healthy 12-year-old male patient was presented to our clinic with right knee pain and functional instability, 1.5 years after a football injury. On physical examination, the knee was unstable with a highly positive Lachman test (Figure 1) and anterior drawer test (Figure 2), there was a mild effusion, collateral ligaments appeared stable, and meniscus signs were negative. MRI revealed a complete tear of the ACL. In addition, an osteochondral lesion of the lateral femoral condyle with a diameter of 1.5 cm was seen. He had been treated nonoperatively with physiotherapy in another facility and was referred due to ongoing pain and giving-way episodes. For his age, the patient was very early in his development (Tanner stage 1) with wide open physes of femur and tibia. After discussing several options with the patient and his parents, we decided to proceed with an all-epiphyseal ACL reconstruction using a quadricep tendon autograft.

## 3. Procedure

**Figure 3:**
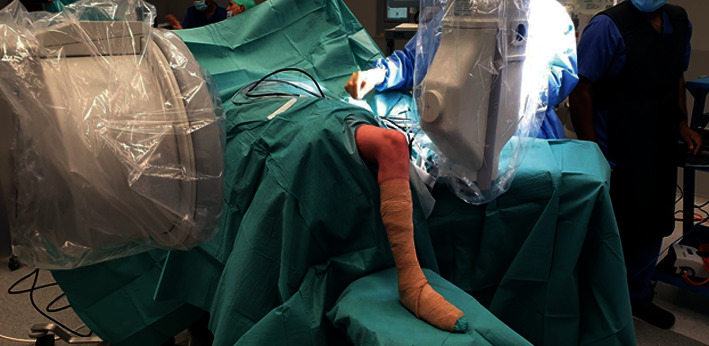
Preoperative position, draping, and preparing.

Under general anesthesia and nerve block, the patient was positioned in supine position on the normal table (Figure 3). A perioperative single-shot antibiotic coverage was given, and a tourniquet was applied.

**Figure 4:**
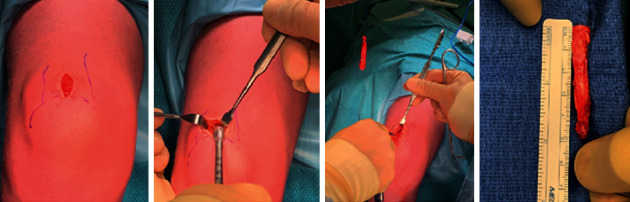
Quadricep tendon autograft was harvested in the minimally invasive technique.

First, a 5 cm × 10 mm × 5 mm quadricep tendon autograft was harvested through a minimally invasive technique (Figure 4) and prepared for an all-inside ACL reconstruction. Here, 15 mm of both ends of the graft was augmented with a fiber loop with fiber tag (FiberLoop w FiberTag Braided Polyblend Blue Suture Loop 20”) (Arthrex) and connected to an ACL tight rope (ACL TIGHTROPE) (Arthrex) .

**Figure 5:**
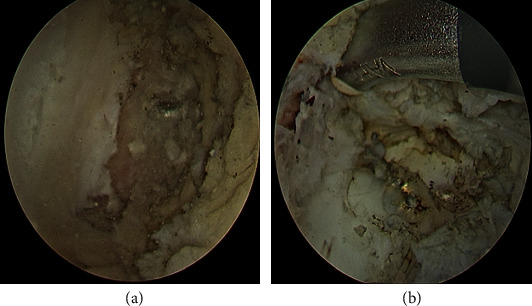
Arthroscopic photo for preparing for tunnels (a) femur drilled tunnel and (b) tibial drilled tunnel.

Then arthroscopic examination of the knee was performed, which revealed a total tear of the ACL. The remnants were debrided, and the femoral and tibial footprints were identified (Figure 5), leaving some tibial fibers in place. Both menisci appeared normal, and the cartilage was unremarkable. Even on the lateral femoral condyle, which demonstrated an osteochondral lesion on MRI, the cartilage appeared stable and did not require any further surgical attention.

**Figure 6:**
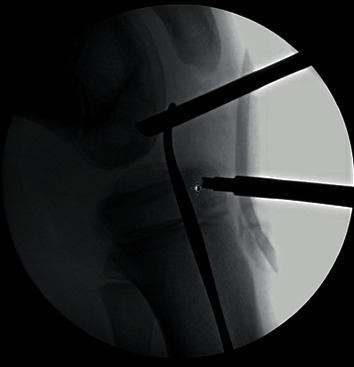
Fluoroscopic image to confirm epiphyseal drilling.

Under fluoroscopy (Figure 6) in true lateral position as well as under direct arthroscopic vision, a guidewire was placed into the exact femoral footprint of the ACL from outside-in. This was over drilled with a 7 mm FlipCutter (FlipCutter II S. A.-N.-1.-7.) (Arthrex) to create the final 1.5 to 2 cm long femoral tunnel. Guiding sutures were pulled through the femoral condyle for later graft placement. During the entire process, it was made sure that the drilling was done strictly epiphyseal.

**Figure 7:**
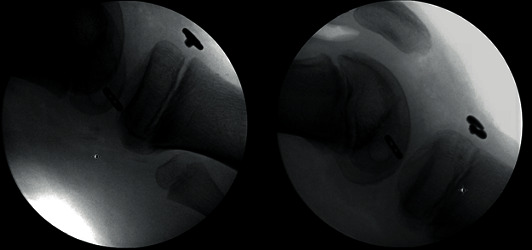
Fixation of the quadricep autograft soft-tissue buttons on both the femur and tibia.

On the tibial side, a guidewire was inserted from the anteriomedial aspect of the proximal tibia into the posterior part of the tibial ACL footprint. It was over drilled with a 6 mm FlipCutter (FlipCutter II S. A.-N.-1.-6.) (Arthrex) in retrograde fashion to create a 1.5 cm tibial tunnel. The drilling again was done under fluroscopic control for a strict epiphyseal position. Guiding sutures were placed in the tibial tunnel and consequently retrieved together with the femoral sutures through the anteriomedial arthroscopic portal. Then, the femoral end of the graft was inserted through the arthroscopic portal, the button flipped on the outside of the lateral femoral condyle, and the graft pulled into the femoral tunnel. The tibial end of the graft was inserted in the similar way, followed by 10 ranges of motion cycles of the knee. Finally, the graft was tensioned in about 20 degrees of flexion. A distal ABS button (TightRope ABS (Attachable Button System)) (Arthrex) was inserted under direct vision to ensure its direct sit on the bone (Figure 7). A final arthroscopic check of the graft and washout of all debris was performed, wounds were closed, and a brace was put on the leg.

The postoperative protocol included early mobilization with weightbearing as tolerated. The child was allowed to remove or wear the brace as wished. Closed chain exercises were initiated early postoperative with special precautions for the weakened quadricep tendon. Physiotherapy continued routinely for 6 weeks postoperatively followed by a guided exercise program. A return to high pivoting activities and competitive sports was only allowed after 9 months.

## 4. Discussion

The anterior cruciate ligament is one of those 4 primary ligaments that connect the femur to the tibia, which is to help restrain mainly the internal rotation and sliding, which will protect the menisci from shearing force that occurs during jumping and deceleration and to give mechanoreceptor feedback to the quadriceps muscle [11] which aids in having a strong and healthy quadriceps muscle. The average ACL length is about 32 mm, and its width is about 9 mm. While the ACL is an intra-articular structure, it is still extra synovial as it is surrounded by a mesentery-like fold of synovium. The ACL is innervated by posterior articular branches of the tibial nerve and is vascularized by branches of the middle genicular branches of popliteal artery; although, the ligament is still hypovascularized and hypocellular. Moreover, disruption of the synovial fold will result in the ACL stumps to float in the synovial fluid and so prevent formation of a local hematoma which is needed to stimulate an inflammatory process and healing, and different tensioning patterns throughout the knee motion, all of this making primary healing impossible. [12].

Usually, an ACL tear is clinically diagnosed, and the Lachman test is the most sensitive, whereas results of the pivot shift test are the most specific [13], but further investigation by MRI is needed to detect any associated injury and to confirm the diagnosis. Historically, pediatric tibial eminence fractures were thought to be more common than ACL tears. However, more recent data suggests that ACL rupture may be more common [14, 15]. In pediatrics, sensitivity and specificity of MRI for detecting ACL tears in children have been reported to be 95% and 88%, respectively. [16].

Increasing in participation in high-demand sports at an earlier age has led to an increase in the rates of ACL tears in the skeletally immature patient. However, the management of an ACL rupture in the skeletally immature patient continues to be a highly controversial topic [17]. In October 2017, the International Olympic Committee hosted an international expert group of physiotherapists and orthopedic surgeons from the United States and Europe who specialize in treating pediatric anterior cruciate ligament (ACL) injuries. The experts supported treating an acute rupture of the ACL surgically with autograft ACL reconstruction if it is associated with other injuries. For those without concomitant injuries, conservative management with high quality rehabilitation to stabilize the knee dynamically as permanent treatment or as short-term option for delayed ACL reconstruction was recommended as well. The group also mentioned that children who undergo ACL reconstruction after failed nonsurgical management may have a higher number of meniscal and chondral injuries at the time of ACL reconstruction compared with those who undergo early ACL reconstruction due to repeated instability episodes, especially if the child receives inadequate or no rehabilitation [18].

A large systematic review including studies from 1986 to 2010 on the topic of treating ACL tears in skeletally immature patients. Vavken et al. reviewed 12 articles on conservative treatment and natural history, 6 of these studies compared conservative with surgical treatment. These reports provide data of 476 patients. They demonstrated poor and unacceptable results for conservative treatment, which commonly leads to meniscal damage and cartilage destruction. They concluded that surgical treatment of the immature patients produces superior clinical outcomes in stability and in the prevention of secondary injury, and conservative treatment should be considered as a last resort [19].) In 2005, Seil and Robert reviewed 17 clinical studies on pediatric ACL injuries and consequent treatment. Conservative management resulted in knee instability in 91% compared to 14% in case of ACL reconstruction [20]. A meta-analysis by Ramski et al. found that children or adolescents undergoing nonoperative or delayed ACL reconstruction were 33.7 times more likely to be clinically unstable and 12 times more likely to subsequently have medial meniscus injury than those who had surgery earlier [21].

Routine primary repair of ACL tears is currently not recommended; although, there has been a recent new interest in primary repair, reserved strictly for acute ruptures of the ACL at its proximal origin and with good stump quality ([22]). However, reconstruction of the ACL is the surgical treatment of choice. Graft selection depends on patient factors and surgeon's preference. The graft can be allograft or autograft. Autograft options usually include a bone-patella tendon-bone (BPTB) autograft, a four-strand hamstring autograft, and a quadricep tendon autograft. Use of allograft for ACL reconstruction in younger, more active patients is not recommended as it is associated with a higher rate of rerupture. [23].

The ultimate tensile load (UTL) of the native ACL is around 2160 N, quadricep tendon graft is around 2353 N, BPTB is around 2977 N, and a quadrupled hamstring autograft is the highest of all graft options at around 4000 N [24].

Hamstring tendon (HT) autograft harvesting carries the risk of weakness of knee flexion and internal rotation, along with injury to branches of the saphenous nerve [25]. In adults, BPTB autografts fuse into the bony tunnels faster than other grafts; so, it is known to be a good choice for patients desiring an early return to sports activity, but it is usually associated with higher donor site morbidity and increased risk for patellar fractures. In the skeletal immature patient, BPTB autografts are usually avoided as transphyseal bone blocks are associated with a higher risk of physeal disruption [26].

The quadricep tendon (QT) autograft was introduced as early as 1979, but has only recently gained increasing popularity as graft of choice for adults and children. The QT shows less anterior knee pain and less risk of sensory deficit [27]. A study comparing QT to BPTB and HT in 2856 patients showed similar rates of graft failure between all groups but did find that QT had less donor site pain than BPTB and better Lysholm scores than HT [28]. Mulford et al. reviewed seventeen articles with a total of 1,580 ACL reconstructions using quadricep autograft and found that it was associated with good clinical and functional outcomes, decreased anterior knee pain, and overall positive outcome [29].

Numerous surgical techniques have been described for ACL reconstruction in the pediatric population. Physeal-sparing techniques (including All-epiphyseal and Over-the-top), combined intra-articular/extra articular by use of autogenous iliotibial band, and traditional adult type transphyseal techniques can be used. The age of the children and the stage of skeletal maturity are the main determinants of which technique to be used. Children below 14 years have open distal femur and proximal tibial physis, which usually complete closure at around 17 years; so, surgeons prefer physeal-sparing techniques for children below 14 years. The physeal-sparing technique avoids injury to the growth plate, but it places the graft in a nonanatomic position which increases the risk of Genu recurvatum [30]. A comparison study between the complications of the tow physeal-sparing technique overgrowth was more common in the all-epiphyseal group and angular deformity in the over-the-top group. Rerupture rates were similar between the group [31], while the transphyseal technique especially with large tunnel diameter shows higher risk of growth disturbance [32]. A larger metanalysis by Wong et al. included 45 articles from 1985 to 2016 on the topic of complications after pediatric ACL reconstruction. The authors concluded that the proper surgical technique is likely more important than the specific reconstruction technique as this will prevent a rerupture of the graft. Growth disturbance can occur after any surgical technique [33]. After reviewing 53 articles, Longo et al. concluded that physeal-sparing techniques had a lower rate of postoperative complications compared with transphyseal techniques, but overall, there is no statistical difference between transphyseal and physeal-sparing techniques [34]. Similar results were found by Pierce et al. in another systematic review [14].

The here presented all-inside, physeal-sparing technique, using quadricep tendon autograft, is a relatively safe procedure with a low morbidity. Using the quadricep tendon decreases the risk of anterior knee pain at the donor site and lowers the risk of nerve injury. The all-inside, physeal-sparing technique avoids the postoperative complications that are associated with other techniques. Several case reports have described this technique as having a good outcome [35] [32]. High-quality rehabilitation is a critical component in the management of ACL injury, and the principles of rehabilitation are the same, irrespective of whether the child has had an ACL reconstruction or has elected for nonsurgical treatment, as this will decrease the rate of secondary injury to the graft after reconstruction. A systematic review showed the increased rate of secondary injury in young athletes who return to sport after ACL reconstruction, which equates to a 30 to 40 times greater risk of an ACL injury compared with uninjured adolescents [36].

## 5. Conclusion

All-inside, all-epiphyseal ACL reconstruction using quadricep tendon autograft in skeletally immature patients is a safe procedure, and it preserves the physeal plate from injury and avoids the graft harvesting procedures seen with other procedure. However, further studies are needed to establish the long-term consequences of this procedure in order to gain wide acceptance in the management of ACL injury in skeletally immature patients.

## Data Availability

The data is available in the hospital database.
